# Lung and abdominal ultrasound accuracy for tuberculosis: An Indian prospective cohort study

**DOI:** 10.1371/journal.pone.0329670

**Published:** 2025-09-03

**Authors:** Stefan Fabian Weber, Rebecca Wolf, Katharina Manten, Balamugesh Thangakunam, Barney Isaac, Deepa Shankar, Divya Mangal, Amit Kumar Dutta, Leena Robinson Vimala, Aparna Irodi, Frank Tobian, Lisa Koeppel, Julia Selena Beck, Peter Wolf, Sabine Bélard, Claudia M. Denkinger, Devasahayam Jesudas Christopher, the ALL POCUS TB India study group

**Affiliations:** 1 Department for Infectious Disease and Tropical Medicine, University Hospital Heidelberg, Heidelberg, Germany; 2 Department for Parasitology, University Hospital Heidelberg, Heidelberg, Germany; 3 German Center for Infectious Disease Research, DZIF Partner Site Heidelberg, Heidelberg, Germany; 4 Department of Anaesthesiology, University Hospital Heidelberg, Heidelberg, Germany; 5 Department of Pulmonary Medicine, Christian Medical College Vellore, Vellore, India; 6 Department of Clinical Gastroenterology, Christian Medical College Vellore, Vellore, India; 7 Department of Radiology, Christian Medical College Vellore, Vellore, India; 8 Department for Pneumology and Critical Care Medicine, Thoraxklinik Heidelberg, Heidelberg, Germany; 9 Institute of Tropical Medicine, University of Tübingen, Tübingen, Germany; 10 German Center for Infectious Disease Research, DZIF Partner Site Tübingen, Tübingen, Germany; Hangzhou Red Cross Hospital, CHINA

## Abstract

**Background:**

Tuberculosis (TB) diagnosis remains a challenge, particularly in low-resource settings. Point-of-care ultrasound (POCUS) has shown promise, but most studies focus on HIV-infected populations. In the case of TB, data on lung ultrasound (LUS) are sparse. Therefore, this study evaluates the diagnostic accuracy of lung and abdominal ultrasound for TB diagnosis in an Indian tertiary care hospital

**Methods:**

We prospectively enrolled adults with presumed TB and performed comprehensive ultrasound assessments. Accuracy of individual and combined sonographic findings was evaluated against a robust reference standard (mycobacterial culture and PCR). Comparators were C-reactive protein at a cut-off of 5mg/l and chest x-ray (CXR). A multivariable model incorporating clinical and ultrasound findings was explored using generalized mixed models and a random forest approach. (Trial registry DRKS00026636).

**Findings:**

Among 541 participants, 102 (19%) were diagnosed with TB and 1% were HIV-positive. The “Focused Assessment with Sonography for HIV-associated TB” (FASH) demonstrated moderate sensitivity (51%) and specificity (70%). Consolidations <1 cm on LUS showed high sensitivity (93%) but low specificity (16%) and were also seen in non-TB lung infections and other conditions like bronchial asthma and COPD. Accuracy of larger (≥1 cm) consolidations (72% sensitive, 55% specific) on LUS was comparable with CXR suggesting possible TB (81% sensitive, 58% specific). Predictive modeling suggests moderate diagnostic performance (AUC = 0.79).

**Interpretation:**

In our study, POCUS did not meet WHO targets for a stand-alone facility-based screening test. Nevertheless, diagnostic accuracy for some findings is comparable to CXR and could be integrated into diagnostic algorithms to improve TB screening where CXR cannot reach. Future research should explore artificial intelligence to enhance TB-POCUS accuracy and accessibility, as was previously reported for CXR.

**Research in context:**

Prior to this study, lung ultrasound (LUS) for TB had been assessed in only a few studies, limited by uncertain sonographic characterization of TB-related findings, lack of consistent terminology, and small numbers of participants with confirmed non-TB diagnoses to determine specificity for TB. Studies evaluating *Focused assessment with sonography for HIV-associated tuberculosis* (FASH) almost exclusively included HIV-infected individuals and demonstrated moderate sensitivity and specificity. However, varying study designs and reference standards limit broader generalization of their findings.

Our prospective study from a TB-endemic setting (India) recruited 541 predominantly HIV-negative participants with presumed TB. This is the largest cohort to date assessing LUS, FASH, and additional ultrasound findings for TB diagnosis. Our study demonstrates that no single ultrasound finding alone, or even in combination, reaches the accuracy targets of the target product profile for a facility-based screening test (triage) proposed by WHO. FASH accuracy in our study aligned with previously reported data in HIV-negative participants but was less specific in HIV-positive participants. The accuracy of additional ultrasound items of LUS and FASH was comparable to chest x-ray (CXR).

In summary, this study demonstrates accuracy of ultrasound for TB diagnosis, backed by a robust study design and using a comprehensive reference standard and CXR comparator for LUS. Modelling suggests that an algorithmic approach combining ultrasound and clinical findings may be of highest value to inform risk of TB and guide further testing to confirm the diagnosis of TB.

Other use cases of POCUS, which may aid clinical decision making in the assessment of disease severity, sampling strategy, and monitoring, should be evaluated by future studies. These should also focus on the accuracy of POCUS in people living with HIV and children, as well as evaluate POCUS more broadly as part of a diagnostic algorithm and by using artificial intelligence to improve the yield of TB-POCUS.

## Introduction

Tuberculosis (TB) diagnosis remains challenging, as highlighted by the diagnostic gap of 26% (global TB incidence 2022: 10·6 million, notified cases 7·8 million) [1]. Appropriate treatment requires access to a reliable diagnostic test and, therefore, the World Health Organization (WHO) demands from diagnostic tests to meet target product profiles. For example, a TB facility-based screening test (triage) should meet a minimum accuracy of 90% sensitivity and 70% specificity [2]. Chest x-ray (CXR) has become more accessible with ultra-portable CXR systems [3] assisted by computer assisted detection using artificial intelligence [4], but high costs and infrastructure needs limit its availability.

Point-of-care ultrasound (POCUS) has emerged as an addition to the diagnostic toolbox [5]. “*Focused Assessment with Sonography for HIV-associated TB”* (FASH) is a protocol designed for HIV-related TB in resource-constrained settings. It screens for pericardial and pleural effusion, hepato-splenic micro-abscesses, and abdominal lymphadenopathy [6]. However, data on accuracy in HIV-uninfected populations are scarce and limited by suboptimal study design: the reported sensitivity ranged between 36–39% and specificity between 70–89% [7–9].

An alternative diagnostic test is lung ultrasound (LUS), and “subpleural nodules” (SUNs) and “miliary pattern” have been associated with pulmonary TB (PTB) [10,11]. A systematic review reported high sensitivity (e.g., SUNs up to 97%). Yet, specificity was not clearly determined due to a lack of adequate control groups and variable ultrasound definitions [12]. Other possible POCUS targets, such as internal mammary lymph nodes (IMNs), mediastinal lymph nodes [13–15], or peritoneal changes [16], have only been described in case studies or series.

This study thus aims to investigate the accuracy of FASH, LUS, and other novel ultrasound targets in a prospective study, testing a representative population in a high TB burden setting comprehensively and against a rigorous reference standard.

## Methods

### Study design and participants

We conducted a single-center, prospective diagnostic accuracy study at an Indian referral hospital (Christian Medical College (CMC), Vellore). Inpatients and outpatients were screened for inclusion criteria by chart review in the Departments of Pulmonary Medicine and Gastroenterology. We consecutively recruited adults ≥18 years with presumed TB disease undergoing a TB workup, who were positive in the WHO TB four-symptom screen: cough ≥2 weeks (any duration in HIV), fever, weight loss, night sweats [17]. Exclusion criteria were microbiological TB confirmation or anti-TB therapy (ATT) initiation before screening, as well as any TB-active medication within the previous six months.

The study was approved by CMC Vellore and University Heidelberg institutional review boards (CMC IRB 14342; Heidelberg S-314/2021) and carried out according to Good Clinical Practice (GCP) guidelines and the Helsinki declaration. Written informed consent was obtained from all participants. The study was registered in the German trial registry (DRKS00026636) and conforms to the Standards for Reporting of Diagnostic Accuracy Studies (STARD, checklist in S1 Supplement) [18].

### Procedures

A standardized questionnaire recorded medical history, clinical, and demographic data. Laboratory tests included HIV-serology (defined as positive if positive current or self-reported prior positive test), HbA1c (diabetes positive if HbA1c ≥ 6·5% or self-reported prior diagnosis) and C-reactive protein (CRP).

At least two respiratory samples (sputum and/or bronchoalveolar lavage (BAL)) were tested with liquid mycobacterial culture (BD BACTEC™ MGIT™ 960); at least one respiratory sample and one spot urine sample (>30ml, centrifuged) were tested with PCR (Xpert® MTB/RIF Ultra). Repeat sputum testing was offered if initial TB tests were negative, no empirical ATT was initiated, and symptoms did not resolve after at least two months.

Discharge information was collected from hospital records for alternative diagnoses in unlikely TB.

### Index test (Table 1, also published in [19])

We used an Edge II (Fujifilm Sonosite, Bothel, United States) with rC60xi (curved array probe), L38xi (linear probe) and rP19x (phased array) probes. FASH: The pre-defined study POCUS protocol included FASH_original_ as published [6]. In addition, we added pericardial, ascites, and pleural effusion measurements [20] to assess the accuracy for a lower pericardial effusion cut-off (FASH_pericardium_), an added pleural effusion cut-off (FASH_pleura_), or presence of ascites (FASH_ascites_). The different variations are contrasted in Table 1. For FASH we used the curved array probe with the “abdominal” preset, the participants were in supine position except for pleural effusions, for which the participant was asked to sit upright.

**Table 1:** Ultrasound findings definitions and FASH variations.

	Definition	Further characteristics assessed	Location
SPC	Subpleural consolidations, hypoechoic or mixed-echoic lesions originating from the visceral pleura	maximum vertical dimension or translobar if not measurable, bronchograms	14 lung zones
B-lines	Vertical lines originating from visceral pleura and extending to >50% of view field, minimum of 3 per lung zone	NA	14 lung zones
Miliary pattern	B-lines, comet artefacts, pleural irregularities, small subpleural consolidations affecting the majority of lung fields	NA	14 lung zones
Pleural thickening	Thickening of parietal or peritoneal pleura	Laminar thickening or nodular thickening, maximum thickness	14 lung zones
Pleural fluid	Content of hypoechoic or mixed echoic echogenicity between parietal and visceral pleura	Echogenicity, measurement of basal lung to diaphragm distance and maximum cranio-caudal distance, estimation of volume (Goecke et al., 1990)	Bilateral pleural recessus
Peritoneal thickening	Thickening of the peritoneal lining in the parietal, visceral or omental layers	Maximum thickness, inclusion of hypoechoic lesions, laminar or nodular thickening	4 abdominal quadrants, transcostal liver view
Intestinal thickening	Thickening of the intestinal wall in the terminal ileum region exceeding 4 mm	–	Right lower abdominal quadrant
Peritoneal fluid	Free abdominal fluid	Amount (small, moderate, large), echogenicity, content	Hepato-renal, spleno-renal and retrovesical pouch as well as inter-intestinal
Pericardial fluid	Pericardial fluid ≥4 mm	Measurement, echogenicity, content	Sub-xiphoidal view
Spleen lesions	Intra-parenchymal spleen lesions -Hypoechoic <1·5 cm -Hypoechoic ≥1·5 cm -echogenic	Measurement, count	Left flank
Liver lesions	Intra-parenchymal hypoechoic lesions	Measurement, count	Subcostal, transcostal
Abdominal lymph nodes	Lymph nodes ≥1·5 cm any dimension	Measurement, architecture, bulking	Liver and spleen hilum, peri-pancreatic, para-aortic
Internal mammary lymph nodes	Lymph nodes ≥0·5 cm, any dimension	Measurement, side, architecture, bulking	Parasternal intercostal spaces
Mediastinal lymph nodes	Lymph nodes ≥1·5 cm, any dimension	Measurement, side, architecture, bulking	Suprasternal view, parasternal view
FASH_original_	-abdominal lymph nodes ≥1·5 cm -hypoechoic liver or spleen lesions -pleural effusion, any amount -pericardial effusion ≥1 cm		
FASH_ascites_	-abdominal lymph nodes ≥1·5 cm -hypoechoic liver or spleen lesions -pleural effusion, any amount -pericardial effusion ≥1 cm -ascites, any amount		
FASH_pericardium_	-abdominal lymph nodes ≥1·5 cm -hypoechoic liver or spleen lesions -pleural effusion, any amount -pericardial effusion ≥0·4 cm		
FASH_pleural_	-abdominal lymph nodes ≥1·5 cm -hypoechoic liver or spleen lesions -pleural effusion, minimum amount specified -pericardial effusion ≥1 cm		

SPC, subpleural consolidations; FASH, focused assessment with sonography for HIV-associated tuberculosis.

Underlined aspects highlight differences to original FASH protocol

NA, not applicable.

This Table was originally published under Creative Commons CC BY (Weber et al., Open Forum Infectious Diseases, 2024, https://doi.org/10.1093/ofid/ofae651).

LUS: Participants were asked to sit upright and we assessed all 14 lung zones vertically and parallel to the intercostal spaces with a linear probe (L38xi) in “lung” preset and a curved probe in case of poor visualization using sweeping motions (Supplement Figure 1 in S1 Supplement) [21]. We assessed each zone for pre-defined findings: A-lines, B-lines, pleural effusions, subpleural consolidations (SPCs), and miliary pattern. For exploratory analyses, we recorded the number, size, and location of findings.

We assessed for parasternal IMNs, peritoneal or omental thickening (all abdominal quadrants), and ileocecal thickening >4mm using the linear probe in “small parts” preset. Using a phased array probe (rP19x), we attempted to visualize mediastinal structures for lymphadenopathy (suprasternal notch and left parasternal view) in “abdominal” preset. In clinically presumed peripheral TB-lymphadenopathy, we scanned respective areas using the linear probe in the “small parts” preset.

Ultrasound was performed on the day of enrollment by non-radiologist clinicians (RW, KM) trained by SFW and blinded to reference standard testing and other clinical data with the exception of the study questionnaire; image acquisition included not only still images, but clips for all views for each participant. Inadequately visualized views were marked non-evaluable. To assess inter-rater agreement and proficiency, a random sample of 15% of participant clips from all sites were evaluated by an additional rater (SFW, RW, KM) regarding SPC_≥1 cm_, SPC_<1 cm_, and FASH items.

### Comparator tests

CXRs acquired within two weeks of recruitment were interpreted by two senior radiologists (blinded to clinical and index test data, consensus read) as *CXR suggesting likely TB* or *CXR suggesting possible TB* as well as *suggestive of post-TB sequelae* (with or without active TB) or *not suggestive of active TB,* guided by published suggestions [22]. CRP was evaluated at a 5 mg/l cut-off [17].

### Reference standard and case definitions

Participants with at least two respiratory samples with TB-culture (pooled or separate), TB-PCR, and urine TB-PCR were assigned to the per protocol (PP) population. Participants with at least one respiratory sample with TB-culture or at least two respiratory samples with TB-PCR, or participants with presumed exclusive extra-pulmonary TB with only non-respiratory samples were assigned to the intention-to-test (ITT) population. Other participants were excluded. We compared PP and ITT by means of a generalized linear mixed model with ‘belonging to PP’ as binary covariate and a random effect accounting for individual variability.

TB disease was diagnosed per microbiological reference standard (MRS), extended MRS (eMRS), or composite reference standard (CRS): i) the MRS was positive if any respiratory or urine sample was positive for *Mycobacterium tuberculosis complex* (MTB) by culture or PCR; ii) the eMRS was positive if either MRS was positive or any other sample was positive for MTB on culture or PCR; iii) the CRS was positive if either MRS or eMRS were positive or empirical ATT was started on clinical grounds with documented clinical improvement. Trace positive PCR results were only considered if a repeat sample was also at least trace positive.

Participants were considered unlikely to have TB if MRS, eMRS, and CRS were negative AND if i) follow-up sputum result was negative, or ii) symptoms resolved, or iii) a plausible alternative diagnosis was made (pre-defined list, see Statistical Analysis Plan). An outcome committee (two senior Indian (DJC, BT) and two senior German (CMD, PW) physicians) with access to clinical and diagnostic data but blinded to the index test assessed remaining cases per consensus ruling as either positive CRS, unlikely TB, or unclassifiable (excluded).

### Statistics

The target sample size was 577 (inputs: sensitivity 60%, specificity 85%, prevalence 20%, precision 10%, dropout 20%) [23]. The primary outcome was POCUS sensitivity and specificity with 95%-confidence intervals (95%-CI) using CRS as primary reference standard. Exploratory outcomes included individual and combined analyses of POCUS findings and subgroup analyses by diabetes and HIV status.

Descriptive data are presented as numbers, proportions, or median and interquartile range (IQR). To explore predictive performance of the data in an HIV-negative cohort, we applied subset selection and machine learning algorithms. First, we excluded all HIV-infected participants and reduced dependency structures within the predictors using factor analysis. Then, we excluded correlating variables carrying similar information to obtain a subset of ultrasound and clinical variables. Statistically significant predictors for TB status were determined using Lasso Regression to punish less impactful predictors. To account for correlations within the predictors, we applied a random forest approach with five-fold cross validation to produce two prediction metrics: AUC (area under the curve) and ROC (Receiver Operating Characteristic) curve. Performance metrices were compared on the subsets of predictors following factor analysis and Lasso regression. Sensitivity analysis included CXR as an additional predictor for both subsets.

We used Cohen’s kappa and a generalized linear mixed model for different rater combinations and random error due to repeated measurements of individuals for the probability of agreement. Statistical analyses were performed using R v.4.2.2 (packages: openxlsx, REDCapR, ggplot2, dplyr, Hmisc) and Python (Version 3.6, libraries: factor_analyzer, statsmodels, sklearn). We used RedCap (Version R 4.2.2 [24]) for data collection. Figures were created using biorender.com, R packages, and the matplotlib Python library.

## Results

**Fig 1:**
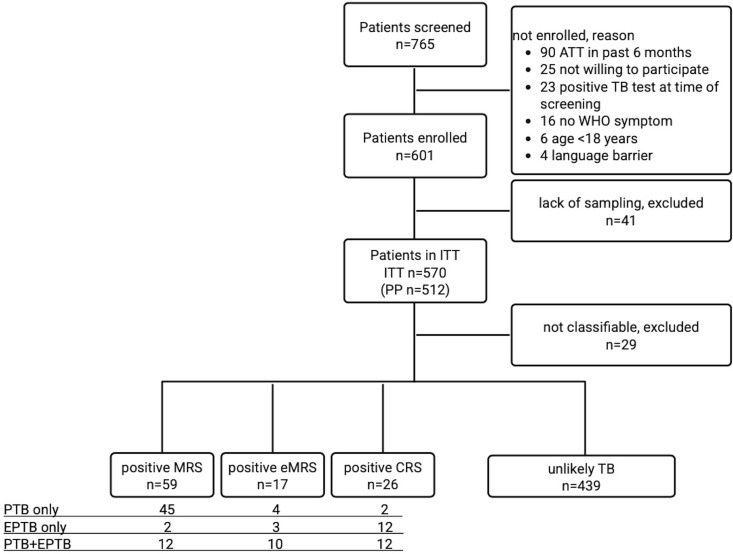
Flow chart for study recruitment and reference standard categories. ATT anti-tuberculous treatment. WHO symptom: cough >2 weeks, fever, night sweats, weight loss. WHO World Health Organization. PP per protocol cohort. ITT intention-to-test cohort. MRS microbiological reference standard. eMRS extended MRS. CRS composite reference standard.

Between 18 April 2022 and 29 July 2023, we screened 765 patients and enrolled 601 participants: 512/601 (85%) were included in the PP and 570/601 (95%) in the ITT cohort, 31/601 (5%) were not in the ITT and excluded. Another 29/601 (5%) could not be assigned to a reference standard and were excluded. In the following, we report results for 541/601 (90%) participants in the ITT cohort (Fig 1).

The generalized linear mixed model revealed that belonging to PP did not significantly affect TB diagnosis (p = 0·987). No relevant differences in index test accuracy for FASH and SPC were observed between groups (PP data in Supplement Table 1 and 2 in S1 Supplement). Thus, we report only on the larger ITT cohort below.

The median age was 48 years, 357/541 (66%) participants were male. Diabetes was present in 121/512 (24%) and HIV in 6/523 (1%). History of prior TB was reported by 99/540 (18%). 483/541 (89%) reported cough, 48/541 (9%) night sweats, 230/541 (43%) fever, and 292/541 (54%) weight loss (Table 2; for MRS and eMRS data and additional information on TB contact history, preexisting comorbidities and symptom duration, see Supplement Table 3 in S1 Supplement).

**Table 2:** Patient characteristics and reference standard testing.

Variables in n, median, IQR, (%)		All participants (n = 541)	CRS positive (n = 102)	Unlikely TB (n = 439)
Age in years		48 [35;59] (N = 541)	44 [29;56] (N = 102)	49 [36;60] (N = 439)
Sex male		357/541 (66%)	65/102 (64%)	292/439 (67%)
Body mass index (kg/m^2)		21·6 [18·4;24·9] (N = 541)	19·4 [17·1;23·1] (N = 102)	22 [19·1;25·2] (N = 439)
Final diabetes status		121/512 (24%)	23/94 (24%)	98/418 (23%)
Final HIV status		6/523 (1%)	1/101 (1%)	5/422 (1%)
History of previous TB disease		99/540 (18%)	11/102 (11%)	88/438 (20%)
History of current TB-contact		5/535 (1%)	3/102 (3%)	2/433 (0%)
Symptoms				
Cough		483/541 (89%)	89/102 (87%)	394/439 (90%)
Hemoptysis		134/541 (25%)	18/102 (18%)	116/439 (26%)
Night sweats		48/541 (9%)	16/102 (16%)	32/439 (7%)
Fever		230/541 (43%)	68/102 (67%)	162/439 (37%)
Weight loss		292/541 (54%)	74/102 (73%)	218/439 (50%)
Fatigue		348/541 (64%)	70/102 (69%)	278/439 (63%)
Loss of appetite		249/541 (46%)	66/102 (65%)	183/439 (42%)
Abdominal pain or distension		89/541 (16%)	24/102 (24%)	65/439 (15%)
Peripheral lymph node swelling		6/541 (1%)	4/102 (4%)	2/439 (0%)
Test results				
C-reactive protein (mg/l)		6 [3;24] (N = 479)	26 [10;58] (N = 91)	4 [3;17] (N = 388)
C-reactive protein >5mg/l*		258/479 (54%)	75/91 (82%)	183/388 (47%)
CXR suggesting likely TB**		80/532 (15%)	34/100 (34%)	46/432 (11%)
CXR suggesting possible TB (includes likely)***		263/532 (49%)	81/100 (81%)	182/432 (42%)
CXR suggestive of post-TB (with or without signs of active TB)		143/527 (27%)	25/97 (26%)	118/430 (27%)
TB-PCR on urine positive		6/519 (1%)	6/101 (6%)	0/418 (0%)
Number of sputa investigated for TB (<2; ≥ 2 samples)		45/541 (8%); 496/541 (92%)	13/102 (13%); 89/102 (87%)	32/439 (7%); 407/439 (93%)
Sputum smear status (% where done)	Negative	488/507 (96)	78/94 (83)	410/413 (99)
	scanty	3/507 (1)	3/94 (3)	0/413
	1+	4/507 (1)	3/94 (3)	1/413 (<1)
	2+	8/507 (2)	6/94 (6)	2/413 (<1)
	3+	4/507 (1)	4/94 (4)	0/413
	not done	25	4	21
Number of patients for whom bronchoalveolar lavage was investigated for TB		157/541 (29%)	30/102 (29%)	127/439 (29%)
Number of patients for whom non-sputum/non-BAL samples were investigated for TB		206/541 (38%)	65/102 (64%)	141/439 (32%)
Positive TB-PCR or culture on sputum or BAL		51/541 (9%)	51/102 (50%)	0/439 (0%)
Positive TB-PCR or culture on non-sputum/non-BAL sample		25/541 (5%)	25/102 (25%)	0/439 (0%)
PTB only EPTB only concurrent PTB+EPTB (%)		NA	51/102 (50) 17/102 (17) 34/102 (33)	NA

Denominators provided for all individuals with available data for each line.

* sensitivity 82% (95% confidence interval (CI) 73–89), specificity 53% (95% confidence interval 48–58).

** sensitivity 34% (95%-CI 25–44); specificity 89% (95%-CI 86–92).

*** sensitivity 81% (95%-CI 72–87); specificity 58% (95%-CI 53–62).

IQR, interquartile range; n, number; MRS, microbiological reference standard; eMRS, extended MRS; CRS, composite reference standard; TB, tuberculoisis; WHO, World Health Organization; HIV, human immunodeficiency virus; ART, anti-retroviral therapy; CXR, chest x-ray; PCR, polymerase chain reaction; BAL, broncho-alveolar lavage; EPTB, extra-pulmonary tuberculosis; NA, not applicable.

TB was diagnosed in 102/541 (19%) participants: 59 with MRS, 17 with eMRS, and 26 with CRS; 439/541 (81%) were deemed unlikely to have TB. TB was limited to PTB only in 59/102 (58%) cases, limited to EPTB (no PTB) in 17/102 (17%) cases, and concurrent EPTB with PTB was present in 26/102 (25%) participants. Common alternative diagnoses were non-TB lung infections (102/439, 23%), asthma (92/439, 21%), and chronic obstructive pulmonary diseases (71/439, 16%) (Table 3).

**Table 3:** Differential diagnoses.

Positive findings	In TB cases (CRS+) n = 102	All unlikely TB	Non-TB lung infection	Asthma	COPD or other obstructive lung disease	Post-TB sequelae	Lung neoplasia	bronchiectasis	ILD	Post-infectious sequelae	Inflammatory bowel disease	Chronic kidney disease	Non-infectious, non-inflammatory gastrointestinal disorder	Systemic autoimmune disorders	Heart disease	Hematological malignancy	Non-pulmonary primary malignancy	Pulmonary arterial hypertension incl. embolic	Gastrointestinal infections	Liver cirrhosis	Cholecystitis	Appendicitis	Tonsillitis	Pulmonary echinococcosis	gingivitis	Pleural empyema
N unlikely TB	NA	439	102	92	71	51	48	44	27	25	17	14	14	12	11	9	8	7	4	2	1	1	1	1	1	1
FASH_original_	52	130	28	15	18	8	31	7	9	4	1	9	1	8	9	5	5	5	1	2	0	0	0	0	0	1
Pleural effusion, any	47	125	28	13	17	8	31	7	8	3	1	9	1	8	9	4	5	5	1	2	0	0	0	0	0	1
Pleural effusion >400ml	42	68	14	2	6	4	25	1	2	0	0	6	0	2	7	3	3	3	0	2	0	0	0	0	0	1
Pericardial effusion >1 cm	2	6	1	1	0	0	3	0	0	0	0	1	0	0	0	0	1	0	0	0	0	0	0	0	0	0
Hypoechoic spleen lesions	4	3	0	0	1	0	0	0	1	0	0	1	0	1	0	2	0	0	0	0	0	0	0	0	0	0
Liver lesions	0	5	1	1	0	0	0	0	2	0	0	1	0	1	0	0	2	2	0	0	0	0	0	0	0	0
Abdominal lymph nodes	7	8	1	0	1	0	1	0	1	1	0	2	0	2	0	3	0	0	0	1	0	0	0	0	0	0
Ascites	14	16	3	1	3	1	1	1	0	0	1	6	2	1	2	1	1	1	0	1	0	0	0	0	0	0
Internal mammary lymph nodes	16	18	12	3	1	4	2	2	0	1	0	1	0	0	0	1	0	0	0	0	0	0	0	0	0	1
Peritoneal thickening	11	3	0	0	0	0	1	0	0	0	0	1	0	0	0	0	0	0	0	1	0	0	0	0	0	0
SPC_ < 1 cm_	95	369	96	71	67	49	43	42	26	22	8	13	9	11	10	9	8	6	4	2	1	0	1	1	1	1
SPC_ ≥ 1 cm_	73	199	67	24	32	38	35	20	19	10	0	9	3	6	6	7	5	4	1	1	0	0	0	1	1	1
Miliary pattern	13	50	15	4	11	11	7	2	14	2	0	3	0	2	3	2	1	3	0	0	0	0	0	0	0	0
CXR suggesting likely TB	34	46	24	4	5	7	5	7	7	3	0	0	0	1	2	3	0	1	0	2	0	0	0	0	0	1
																						highest value color			102	
																						lowest value color			0	

TB, tuberculosis; n, number; ILD, interstitial lung disease; COPD, chronic obstructive pulmonary disease; FASH, focused assessment with sonography for HIV-associated tuberculosis; SPC, subpleural consolidations; CXR, chest x-ray.

### Index test

FASH_original_ sensitivity was 51% (95%-CI 41–60) and specificity 70% (95%-CI 66–74). Sensitivity was mostly driven by pleural effusions (47/102, 46%, of TB cases), other FASH-components were rare: abdominal lymph nodes were present in 15/541 (3%) participants, pericardial effusions in 8/541 (1%) participants, hypoechoic spleen lesions in 7/541 (1%, cf. Supplement Figure 2C in S1 Supplement) participants, and hypoechoic liver lesions in 5/541 (1%) participants (Table 4).

**Table 4:** POCUS – lung ultrasound, FASH, exploratory targets.

	All participants (n = 541)	CRS positive (n = 102)	Unlikely TB (n = 439)	Sensitivity (95%-CI)	Specificity (95%-CI)
FASH					
FASH_original_	182/541 (34%)	52/102 (51%)	130/439 (30%)	0·51 [0·41;0·6]	0·7 [0·66;0·74]
FASH_ascites_	187/541 (35%)	53/102 (52%)	134/439 (31%)	0·52 [0·42;0·61]	0·69 [0·65;0·74]
FASH_pericardium_	196/541 (36%)	54/102 (53%)	142/439 (32%)	0·53 [0·43;0·62]	0·68 [0·63;0·72]
FASH_pleura600ml_	105/541 (19%)	36/102 (35%)	69/439 (16%)	0·35 [0·27;0·45]	0·84 [0·81;0·87]
Pleural effusion present, any	172/541 (32%)	47/102 (46%)	125/439 (28%)	0·46 [0·37;0·56]	0·72 [0·67;0·76]
Pleural effusion volume estimate	637 [288;1262] (N = 172)	914 [513;1483] (N = 47)	462 [256;1160] (N = 125)	NA	NA
Pericardial effusion ≥10 mm	8/541 (1%)	2/102 (2%)	6/439 (1%)	0·02 [0·01;0·07]	0·99 [0·97;0·99]
Hypoechoic spleen lesions <1·5 cm present	7/541 (1%)	4/102 (4%)	3/439 (1%)	0·04 [0·02;0·1]	0·99 [0·98;1]
Hyperechoic spleen lesions present with or without calcification	23/540 (4%)	5/102 (5%)	18/438 (4%)	NA	NA
Hypoechoic liver lesions	5/541 (1%)	0/102 (0%)	5/439 (1%)	0 [0;0·04]	0·99 [0·97;1]
Abdominal lymph nodes ≥1·5 cm present	15/541 (3%)	7/102 (7%)	8/439 (2%)	0·07 [0·03;0·13]	0·98 [0·96;0·99]
Ascites present	30/541 (6%)	14/102 (14%)	16/439 (4%)	0·14 [0·08;0·22]	0·96 [0·94;0·98]
LUNG ULTRASOUND					
Subpleural consolidations (SPC) <1 cm present	464/541 (86%)	95/102 (93%)	369/439 (84%)	0·93 [0·87;0·97]	0·16 [0·13;0·2]
SPCs with regular round/oval shape, hypoechoic echogenicity and posterior enhancement	12/541 (2%)	1/102 (1%)	11/439 (3%)	0·01 [0;0·05]	0·97 [0·96;0·99]
>5 SPCs_ < 1 cm_ with at least one ≥ 5 mm	150/541 (28%)	32/102 (31%)	118/439 (27%)	0·31 [0·23;0·41]	0·73 [0·69;0·77]
Subpleural consolidations ≥1 cm present	272/541 (50%)	73/102 (72%)	199/439 (45%)	0·72 [0·62;0·79]	0·55 [0·5;0·59]
any subpleural consolidation present, regardless of size	476/541 (88%)	100/102 (98%)	376/439 (86%)	0·98 [0·93;0·99]	0·14 [0·11;0·18]
any consolidation in the apical regions	294/541 (54%)	66/102 (65%)	228/439 (52%)	0·65 [0·55;0·73]	0·48 [0·43;0·53]
any consolidations <1 cm in the apical regions	252/541 (47%)	57/102 (56%)	195/439 (44%)	0·56 [0·46;0·65]	0·56 [0·51;0·6]
any consolidations ≥1 cm in the apical regions	84/541 (16%)	22/102 (22%)	62/439 (14%)	0·22 [0·15;0·31]	0·86 [0·82;0·89]
miliary pattern present	63/541 (12%)	13/102 (13%)	50/439 (11%)	0·13 [0·08;0·21]	0·89 [0·85;0·91]
LUS findings in at least … lung zones					
SPC_ < 1 cm_ in at least 1 lung zone	464/541 (86%)	95/102 (93%)	369/439 (84%)	0·93 [0·87;0·97]	0·16 [0·13;0·2]
≥2 lung zones	390/541 (72%)	84/102 (82%)	306/439 (70%)	0·82 [0·74;0·89]	0·3 [0·26;0·35]
≥3 lung zones	315/541 (58%)	72/102 (71%)	243/439 (55%)	0·71 [0·61;0·79]	0·45 [0·4;0·49]
≥4 lung zones	247/541 (46%)	52/102 (51%)	195/439 (44%)	0·51 [0·41;0·6]	0·56 [0·51;0·6]
≥5 lung zones	186/541 (34%)	38/102 (37%)	148/439 (34%)	0·37 [0·28;0·47]	0·66 [0·62;0·71]
≥6 lung zones	146/541 (27%)	22/102 (22%)	124/439 (28%)	0·22 [0·15;0·31]	0·72 [0·67;0·76]
≥7 lung zones	114/541 (21%)	16/102 (16%)	98/439 (22%)	0·16 [0·1;0·24]	0·78 [0·74;0·81]
≥8 lung zones	83/541 (15%)	15/102 (15%)	68/439 (15%)	0·15 [0·09;0·23]	0·85 [0·81;0·88]
≥9 lung zones	53/541 (10%)	11/102 (11%)	42/439 (10%)	0·11 [0·06;0·18]	0·9 [0·87;0·93]
SPC_ ≥ 1 cm_ in at least 1 lung zone	272/541 (50%)	73/102 (72%)	199/439 (45%)	0·72 [0·62;0·79]	0·55 [0·5;0·59]
≥2 lung zones	194/541 (36%)	57/102 (56%)	137/439 (31%)	0·56 [0·46;0·65]	0·69 [0·64;0·73]
≥3 lung zones	129/541 (24%)	39/102 (38%)	90/439 (21%)	0·38 [0·29;0·48]	0·79 [0·75;0·83]
≥4 lung zones	86/541 (16%)	27/102 (26%)	59/439 (13%)	0·26 [0·19;0·36]	0·87 [0·83;0·89]
≥5 lung zones	52/541 (10%)	13/102 (13%)	39/439 (9%)	0·13 [0·08;0·21]	0·91 [0·88;0·93]
B-lines in at least 1 lung zone	246/541 (45%)	53/102 (52%)	193/439 (44%)	0·52 [0·42;0·61]	0·56 [0·51;0·61]
≥2 lung zones	176/541 (33%)	37/102 (36%)	139/439 (32%)	0·36 [0·28;0·46]	0·68 [0·64;0·73]
≥3 lung zones	120/541 (22%)	24/102 (24%)	96/439 (22%)	0·24 [0·16;0·33]	0·78 [0·74;0·82]
≥4 lung zones	92/541 (17%)	20/102 (20%)	72/439 (16%)	0·2 [0·13;0·28]	0·84 [0·8;0·87]
≥5 lung zones	72/541 (13%)	15/102 (15%)	57/439 (13%)	0·15 [0·09;0·23]	0·87 [0·84;0·9]
≥6 lung zones	51/541 (9%)	10/102 (10%)	41/439 (9%)	0·1 [0·05;0·17]	0·91 [0·88;0·93]
Other targets					
IMNs ≥ 0·5 cm present	34/541 (6%)	16/102 (16%)	18/439 (4%)	0·16 [0·1;0·24]	0·96 [0·94;0·97]
Pleural thickening	44/541 (8%)	14/102 (14%)	30/439 (7%)	NA	NA
Intestinal thickening in the right lower quadrant >4mm	15/530 (3%)	9/102 (9%)	6/428 (1%)	0·09 [0·05;0·16]	0·99 [0·97;0·99]
Any peritoneal thickening	14/541 (3%)	11/102 (11%)*	3/439 (1%)	0·11 [0·06;0·18]	0·99 [0·98;1]
Mediastinal lymph nodes seen from suprasternal view **	2/540 (0%)	0/102 (0%)	2/438 (0%)	NA	NA
Peripheral lymph nodes present (only if clinical suspicion)	3/539 (1%)	1/102 (1%)	2/437 (0%)	NA	NA

Denominators provided for all individuals with available data for each line.

* This affected the omentum in most cases (10/11) with additional hypoechoic nodules in 8/10.

** parasternal only negative.

IQR, interquartile range; n, number; MRS, microbiological reference standard; eMRS, extended MRS; CRS, composite reference standard; TB, tuberculoisis; FASH, focused assessment with sonography for HIV-associated tuberculosis; SPC, subpleural consolidation; LUS, lung ultrasound; IMN, internal mammars lymph node; NA, not applicable.

In order to explore adaptations to FASH, setting a 600 ml threshold for pleural effusions (FASH_pleural600ml_) decreased sensitivity to 35% (95%-CI 27–45) but increased specificity to 84% (95%-CI 81–87), reflecting larger volumes in TB (TB cases median 914 ml vs. unlikely TB 462 ml). Including ascites (FASH_ascites_) or reducing the pericardial effusion cut-off to 4 mm (FASH_pericardium_) did not significantly influence accuracy (Table 4; additional variations: Supplement Table 4 in S1 Supplement). Of 130 FASH_original_-positive cases with unlikely TB, 31/130 (24%) had lung neoplasia and 28/130 (22%) had non-TB lung infections (other alternative diagnoses: Table 3).

On LUS (Supplement Figures 3A-D in S1 Supplement), SPCs_<1 cm_ were common in both TB cases and unlikely TB (sensitivity 93%, specificity 16%). Larger SPCs_≥1 cm_ were less sensitive (72%), but more specific (55%). Exploring the location or number of zones affected by SPCs, we found higher specificity, but lower sensitivity if only considering SPCs_≥1 cm_ in the apical lung zones (sensitivity 22%, specificity 86%) or SPCs_≥1 cm_ in at least three lung zones (sensitivity 38%, specificity 79%).

Miliary pattern was seen with similar frequency in TB cases (13/102, 13%) and unlikely TB (50/439, 11%) (Table 4; further details in Supplement Table 4 in S1 Supplement).

Of 369 SPCs_<1 cm_-positive cases, 96/369 (26%) had non-TB lung infections, 71/369 (19%) had asthma; of 209 SPCs_≥1 cm_-positive cases with unlikely TB, most were associated with non-TB lung infections (67/209, 32%) and post-TB sequelae (38/209, 18%) (Table 3).

Exploratory findings were each present in <20% of participants: prevalence of IMNs (Supplement Figure 2A in S1 Supplement) in TB cases was 16/102 (16%) versus 18/439 (4%) in unlikely TB; prevalence of pleural thickening was 14/102 (14%) in TB cases versus 30/439 (7%) in unlikely TB; peritoneal thickening (c.f., Supplement Figure 2B,D in S1 Supplement) prevalence in TB cases was 11/102 (11%) compared to 3/439 (1%) in unlikely TB; finally, prevalence of intestinal thickening was 9/102 (9%) in TB cases compared to 6/438 (1%) in unlikely TB.

### Subgroup analyses by HIV and diabetes status

Pleural effusions were slightly more common in participants with diabetes (36%) than in those without (31%). There were no relevant differences between groups regarding FASH findings or SPCs_<1 cm_ and SPCs_≥1 cm_ (Table 5). For ultrasound in the HIV-infected subgroup, case numbers were insufficient for analysis (n = 7) (Supplement Table 5 in S1 Supplement).

**Table 5:** POCUS – stratified by diabetes.

	DM-, all (n = 391)	DM-, CRS+ (n = 71)	DM-, unlikely (n = 320)	DM + , all (n = 121)	DM + , CRS+ (n = 23)	DM + , unlikely (n = 98)
CXR suggestive of active TB	54/383 (14%)	24/69 (35%)	30/314 (10%)	24/121 (20%)	9/23 (39%)	15/98 (15%)
CXR suggestive or consistent with active TB	180/383 (47%)	56/69 (81%)	124/314 (39%)	74/121 (61%)	20/23 (87%)	54/98 (55%)
Positive TB-PCR on sputum or BAL	32/391 (8%)	32/71 (45%)	0/320 (0%)	14/121 (12%)	14/23 (61%)	0/98 (0%)
Positive TB-culture on sputum or BAL	28/391 (7%)	28/71 (39%)	0/320 (0%)	10/121 (8%)	10/23 (43%)	0/98 (0%)
Positive TB-PCR on non-sputum/BAL sample	13/391 (3%)	13/71 (18%)	0/320 (0%)	4/121 (3%)	4/23 (17%)	0/98 (0%)
Positive TB-culture on non-sputum/BAL sample	8/391 (2%)	8/71 (11%)	0/320 (0%)	3/121 (2%)	3/23 (13%)	0/98 (0%)
PTB only EPTB only PTB+EPTB	34/71 (48) 14/71 (20) 23/71 (32)			14/23 (61) 1/23 (4) 8/23 (35)		
FASH (original) positive	129/391 (33%)	39/71 (55%)	90/320 (28%)	45/121 (37%)	10/23 (43%)	35/98 (36%)
Pleural effusion present, any	121/391 (31%)	34/71 (48%)	87/320 (27%)	44/121 (36%)	10/23 (43%)	34/98 (35%)
Pericardial effusion ≥10 mm	5/391 (1%)	2/71 (3%)	3/320 (1%)	2/121 (2%)	0/23 (0%)	2/98 (2%)
Hypoechoic spleen lesions <1·5 cm present	5/391 (1%)	3/71 (4%)	2/320 (1%)	1/121 (1%)	1/23 (4%)	0/98 (0%)
Hypoechoic liver lesions	4/391 (1%)	0/71 (0%)	4/320 (1%)	1/121 (1%)	0/23 (0%)	1/98 (1%)
Abdominal lymph nodes ≥1·5 cm present	9/391 (2%)	4/71 (6%)	5/320 (2%)	5/121 (4%)	3/23 (13%)	2/98 (2%)
Ascites present	18/391 (5%)	6/71 (8%)	12/320 (4%)	9/121 (7%)	5/23 (22%)	4/98 (4%)
Subpleural consolidations (SPC) <1 cm present	333/391 (85%)	67/71 (94%)	266/320 (83%)	108/121 (89%)	22/23 (96%)	86/98 (88%)
Subpleural consolidations ≥1 cm present	195/391 (50%)	52/71 (73%)	143/320 (45%)	65/121 (54%)	17/23 (74%)	48/98 (49%)
any subpleural consolidation present, regardless of size?	340/391 (87%)	70/71 (99%)	270/320 (84%)	112/121 (93%)	23/23 (100%)	89/98 (91%)
miliary pattern present	47/391 (12%)	11/71 (15%)	36/320 (11%)	13/121 (11%)	1/23 (4%)	12/98 (12%)
B-lines (>2) in at least one lung zone	182/391 (47%)	40/71 (56%)	142/320 (44%)	49/121 (40%)	8/23 (35%)	41/98 (42%)
Internal mammary lymph nodes (IMNs) ≥0·5 cm present	28/391 (7%)	13/71 (18%)	15/320 (5%)	5/121 (4%)	2/23 (9%)	3/98 (3%)
Pleural nodules or laminar thickening	30/391 (8%)	11/71 (15%)	19/320 (6%)	12/121 (10%)	3/23 (13%)	9/98 (9%)
Intestinal thickening in the right lower quadrant >4mm	9/385 (2%)	6/71 (8%)	3/314 (1%)	2/117 (2%)	1/23 (4%)	1/94 (1%)
Any peritoneal thickening	6/391 (2%)	4/71 (6%)	2/320 (1%)	6/121 (5%)	5/23 (22%)	1/98 (1%)
Peripheral lymph nodes present (only if clinical suspicion)	2/390 (1%)	0/71 (0%)	2/319 (1%)	1/120 (1%)	1/23 (4%)	0/97 (0%)

Denominators provided for all individuals with available data for each line.

DM, diabetes mellitus; n, number; HIV, human immunodeficiency virus; CRS, composite reference standard; CXR, chest x-ray; TB, tuberculosis; PCR, polymerase chain reaction; BAL, broncho-alveolar lavage; EPTB, extra-pulmonary tuberculosis; PTB, pulmonary tuberculosis; FASH, focused assessment with sonography for HIV-associated tuberculosis; SPC, subpleural consolidations.

Interobserver agreement calculations for ultrasound rating yielded an overall Cohen’s kappa of 0·71, the generalized linear mixed yielded a probability of agreement of 98·8%, strongly supporting coherence in rater decisions. No adverse events were observed as part of the reference standard or index test.

### Comparator tests

**Fig 2:**
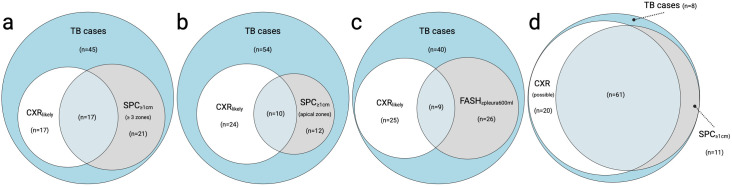
Venn diagrams of overlap between lung ultrasound findings and chest x-ray in tuberculosis cases. a CXRlikely vs. SPC ≥ 1 cm in at least 3 lung zones. b CXRlikely vs. SPC ≥ 1 cm in the apical lung zones. c CXRlikely vs. FASHpleura600ml. d CXRpossible vs. any SPC ≥ 1 cm. Color code: Blue area: TB cases (CRS) with no positive CXR or ultrasound finding. White circle (left): TB cases with positive chest x-ray. Gray circle (right): TB cases with positive ultrasound. Overlap light blue: TB cases with positive ultrasound and chest x-ray. CRS: composite reference standard. CXR: chest x-ray. CXRlikely CXR suggesting likely TB. CXRpossible CXR suggesting possible TB. SPC ≥ 1 cm subpleural consolidation ≥1 cm. FASHpleural600ml: Focused assessment with sonography for HIV-associated tuberculosis with a pleural effusion threshold of 600 ml.

*CXR suggesting likely TB* showed a sensitivity of 34% (95%-CI 25–44) and a specificity of 89% (95%-CI 86–92), comparable to FASH_peural600ml_ which was 35% sensitive and 84% specific. *CXR suggesting possible TB* (includes *CXR suggesting likely TB*) had a sensitivity of 81% (95%-CI 72–87) and specificity of 58% (95%-CI 53–62), which was within the range of any SPCs_≥1 cm_ (72% sensitivity, 55% specificity). CRP > 5 mg/l had a sensitivity of 82% (95%-CI 73–89) and a specificity of 53% (95%-CI 48–58). Venn diagrams comparing POCUS and CXR are provided in Fig 2.

### Combinations of LUS, CXR and CRP

We additionally explored the combined accuracy of LUS findings (SPC_≥1 cm_, SPC_≥1 cm_ in the apical regions and SPC_<1 cm_), *CXR suggesting likely TB* and CRP > 5 mg/l. Neither combination showed a relevant improvement of accuracy, e.g., *CXR suggesting likely TB* or CRP > 5 mg/l was 30% sensitive and 93% specific; *CXR suggesting likely TB* or any SPC_≥1 cm_ was 28% sensitive and 91% specific; CRP > 5 mg/l or any SPC_≥1 cm_ was 61% sensitive and 73% specific. Selected exploratory combinations are provided in Supplement Table 4 in S1 Supplement.

### Predictive modeling analysis

Including all variables (except CXR) derived after the factor analysis reached an AUC of 0·79. Lasso regression led to a reduction from 22 to 10 significant ones, while decreasing the AUC to 0·75 only. SHAP (SHapley Additive exPlanations) value analysis, which quantifies the contribution of each input feature to the model’s predictions, showed that the top five variables (CRP, age, spleen size, pleural effusion volume estimate, symptom fever) were identical across both scenarios. Among these, CRP exhibited by far the greatest impact on prediction outcomes, followed by Age, with a marked decrease in importance observed for the remaining features. Directionality plots and a complete list of variables and their corresponding SHAP value is provided in the S1 Supplement.

**Fig 3:**
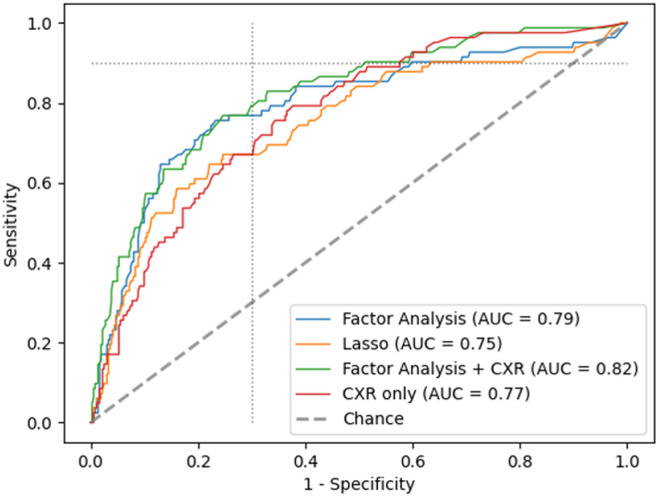
Receiving operator curve (ROC) for ultrasound, chest x-ray and other participant variables for tuberculosis diagnosis. Dotted lines: cut-off for 70% specificity and 90% sensitivity. AUC: area under the curve. CXR: chest x-ray. Variables in factor analysis and Lasso analysis, see in S1 Supplement.

Combining CXR with all variables after factor analysis achieved an AUC of 0.82. Using CXR only, the AUC reached 0·77. Comparing ROC curves, CXR revealed a slightly better performance, especially in the aspects of the ROC curve related to high sensitivity (Fig 3). Further details and all variables used in the model are provided in the S1 Supplement section “Predictive modeling analysis”.

## Discussion

In this study we report the largest prospective cohort investigated for LUS, FASH, and other extrapulmonary POCUS findings in patients with presumed TB. Our study demonstrates limited performance of individual findings of POCUS findings but an accuracy comparable to CXR.

In our predominantly HIV-uninfected cohort, FASH showed moderate sensitivity (51%) and specificity (70%), differing from a previous smaller studies in India (39% sensitivity, 70% specificity [9]) and South African (36% sensitive, 89% specific [8]). Our investigation builds upon these studies not only by its larger cohort but also contributes results which are more generalizable due to an adequate control group. However, our results fare less well than those of a systematic review that included studies irrespective of HIV-status and found a pooled sensitivity of 72% and specificity of 77% for EPTB-ultrasound [25]. This can be attributed to the highly biased populations included in the systemic review.

Generally, performance was lower than in some studies including only HIV-positive cases (e.g., [9]). In our TB cases, larger pleural effusions were wide-spread, contrasting with HIV-infected individuals, where abdominal lymphadenopathy (range 36–86%) and spleen lesions (range 13–62%) were more common [26]. Less frequent EPTB findings may not be relevant in a POCUS use-case for HIV-negative individuals.

Accuracy of FASH_pleural600ml_ was comparable with CXR (*CXR suggesting likely TB*) although it detected different TB-cases (26% of TB-cases detected by FASH_pleural600ml_ but not by *CXR suggesting likely TB*, Fig 2A), which suggests also a complementary value of POCUS to CXR. FASH may also benefit HIV-uninfected individuals by exploring disease spread or targeting diagnostic interventions.

LUS accuracy varied with the size, location, and number of findings investigated. Hypoechoic and regularly oval SPCs_<1 cm_ with posterior enhancement (i.e., suggested SUN [10,11]) were seen only in 12/541 (2%) participants and irregular-shaped “mini shreds” were revealed by linear ultrasound in most cases (cf. Supplement Figure 3A/B in S1 Supplement). SPC_≥1 cm_ in the apical lung zones or SPC_≥1 cm_ in at least three lung zones were in a similar accuracy range as *CXR suggesting likely TB*. Any SPC_≥1 cm_ was slightly less sensitive but comparably specific as *CXR suggesting possible TB* (cf. previous data for CXR in facility-based screening: e.g., 90% sensitivity, 56% specificity [27] or 88% sensitivity, 63% specificity [28]).

Importantly, no LUS finding or variation met WHO target product profile goals [2] as a stand-alone test, but compared with CXR, LUS offers advantages in portability and absence of ionizing radiation. Inclusion of LUS in a diagnostic algorithm may be justified in appropriate settings (e.g., forwarding LUS-positive cases for confirmatory testing) and additional uses may include the assessment of disease severity by quantifying the unaerated lung surface [29].

IMNs, pleural, or peritoneal thickening were rare, but more common in TB cases and if found, may guide clinical decision making and sampling strategies in difficult-to-diagnose cases. Unlike in children [15], where the thymus gland serves as a sound window into the mediastinum for an assessment of mediastinal lymph nodes, this region was not reliably assessable in our adult cohort population in absence of this sound window. An adequate assessment of these nodes would require either cross-sectional imaging or endobronchial ultrasound (EBUS), which is beyond the POCUS use case and highlights a limitation of chest ultrasound for TB.

All LUS and EPTB findings were also found in other lung infections, post-TB sequelae, lung neoplasia, and obstructive lung disorders, limiting its specificity substantially.

Predictive modeling analysis of ultrasound in combination with clinical variables suggests comparable performance of ultrasound and CXR in terms of AUC. We hypothesize that the observed marginal differences in ROC curves may be attributable to random variation, nonetheless, further research is required to validate this observation. However, it must be acknowledged that CXR remains the standard of care in most settings and therefore the potential added value of LUS is context-dependent and may be greatest in primary or secondary care settings where access to CXR is limited or unavailable. Development of a diagnostic algorithm for clinical implementation (with LUS replacing or LUS complementing CXR) will require further work accounting for the inherent correlation structure of predictors and the variety of extrapulmonary TB, followed by an external validation.

## Limitations and strengths

Our study was conducted at a tertiary referral hospital to ensure access to a high-quality diagnostic reference standard; however, this may limit generalizability to lower levels of care. Future POCUS evaluations should therefore prioritize validation in various levels of healthcare. The low prevalence of HIV in our cohort does not allow conclusions to be drawn in HIV-infected populations and further studies should investigate the accuracy and added value of POCUS in an HIV-infected population. We excluded patients with prior TB treatment within the past six months, aiming to reduce confounding of this treatment with POCUS findings. This may have result in an underrepresentation of drug-resistant or recurrent TB cases, but the high proportion (18%) of participants reporting previous TB mitigates concerns about limited generalizability to recurrent cases.

Strengths of our study included the large sample size, the robust reference standard (over 90% with at least two sputa) and follow-up, and the expert committee to optimize case definitions. The index test was comprehensive and video documentation ensured standardization. Reproducibility of ultrasound interpretation was confirmed by a high inter-observer agreement. For CXR reading, blinded review by TB-experienced radiologists enabled a representative comparison. In total, these strengths lead to a high level of generalizability of our data in similar settings.

## Conclusions

While accuracy of LUS, FASH, and other ultrasound findings in HIV-uninfected individuals did not achieve diagnostic performance required for a TB facility-based screening test, its performance is similar to CXR and could serve a complementary role. Combined with the lower cost and easier accessibility of ultrasound, POCUS could enable a broader availability of imaging. Additional use cases for POCUS, such as delineating spread and severity of TB, could also be of substantial value to TB-programs. Further studies should aim to generate evidence in additional populations (e.g., children) and across different clinical use cases. This includes the possible role of ultrasound as part of diagnostic algorithms (e.g., screening at the primary care level). In parallel, developing artificial intelligence-aided ultrasound for detection of abnormalities like subpleural consolidations, may enhance diagnostic accuracy and increase access by reducing the dependence on trained operators.

## Supporting information

S1 Supplement Study protocol: DOI https://doi.org/10.11588/data/KFNN2N. Statistical analysis plan: DOI https://doi.org/10.11588/data/KFNN2N. Data set: DOI https://doi.org/10.11588/data/KFNN2N. Supplement Figure 1 *– schematic of lung zones and probe movement.* Supplement Figure 2*- Panel: extrapulmonary ultrasound examples.* Supplement Figure 3*- Panel: lung ultrasound examples.* Supplement Table 1 Supplement Table 1 Comparison of per protocol (PP) and intention to test (ITT) groups. Supplement Table 2 PP data for cohort characteristics, test results and index test. Supplement Table 3 – patient characteristics and reference standard testing – extended. Supplement Table 4 – POCUS – lung ultrasound, FASH, exploratory targets – extended. Supplement Table 5 – POCUS – stratified by HIV and diabetes. Interrater analysis. Predictive modelling analysis. STARD checklist.
(DOCX)

## Abbreviations

ATT: anti-tuberculous treatment
AUC: area under the curve
BAL: bronchoalveolar lavage
CRP: C-reactive protein
CRS: composite reference standard
CXR: chest x-ray
eMRS: extended microbiological reference standard
EPTB: extra-pulmonary tuberculosis
FASH: Focused Assessment with Sonography for HIV-associated tuberculosis
FASH_ascites_: FASH protocol including ascites (see definitions in Table 1)
FASH_original_: FASH protocol as previously published
FASH_pericardium_: FASH protocol including lower pericardial effusion cut-off (see definitions in Table 1)
FASH_pleura_: FASH protocol including pleural effusion cut-off (see definitions in Table 1)
GCP: good clinical practice
HIV: human immunodeficiency virus
IMN: internal mammary lymph nodes
IQR: interquartile range
ITT: intention to test population
LUS: lung ultrasound
MRS: microbiological reference standard
PCR: polymerase chain reaction
POCUS: point-of-care ultrasound
PP: per protocol population
PTB: pulmonary tuberculosis
ROC: receiver operating characteristic
SPC: subpleural consolidation
SPC<=1 cm: subpleural consolidation 1 cm or larger in size
SPC < 1 cm: subpleural consolidation smaller than 1 cm
STARD: Standards for Reporting of Diagnostic Accuracy Studies
SUN: subpleural nodule
TB: tuberculosis
WHO: World Health Organization
